# Unveiling the association between plasma lipid species and pan-cancers: A bidirectional 2-sample Mendelian randomization study

**DOI:** 10.1097/MD.0000000000050711

**Published:** 2026-09-25

**Authors:** Xiaofeng Cui, Xinguo Yang, Guangxi Piao

**Affiliations:** a Department of Anesthesiology, Jilin Provincial People’s Hospital, Changchun, Jilin, China; b Department of Pathology, The First Hospital of Jilin University, Changchun, Jilin, China.

**Keywords:** cancers, genetic causal association, Mendelian randomization, plasma lipids

## Abstract

Plasma lipids play a crucial role at every stage of cancer progression, and a deficiency or excess of plasma lipids can lead to an increased risk of cancer. Previous observational studies and randomized controlled trials on the association between plasma lipids and cancer risk have been limited. We conducted a Mendelian randomization (MR) analysis to assess the impact of 179 plasma lipid species on the risk of 15 types of cancer. Through bidirectional 2-sample MR analysis and colocalization analysis, we systematically studied the association between 179 plasma lipid species and 15 types of cancer. In the MR study, we primarily used inverse variance weighting as our analytical strategy. Sensitivity analyses were carried out using the MR-Egger method and the MR Pleiotropy Residual Sum and Outlier method. We rigorously assessed heterogeneity through the application of Cochrane *Q* test. To ensure the robustness of the MR results, we employed the “leave-one-out” method and tested the strength of the causal relationships through false discovery rate correction. We identified a significant causal relationship between 20 plasma lipid species and the risk of 5 types of cancer. Specifically, in the forward MR analysis, the level of plasma phosphatidylethanolamine (18:0_20:4) was causally linked with pancreatic cancer; the level of plasma phosphatidylcholine (O-18:0_16:1) was causally linked with breast cancer (estrogen receptor [ER]+); the level of plasma phosphatidylcholine (O-16:0_20:3) was causally linked with endometrial cancer (endometrioid histology); 3 plasma lipid species were causally linked with lung cancer; and 14 plasma lipid species were causally linked with colorectal cancer. Strong evidence from colocalization analysis confirmed that 11 plasma lipid species shared causal variants with the risk of colorectal cancer. In the reverse analysis, an increased risk of colorectal cancer was associated with elevated levels of plasma phosphatidylcholine (18:1_18:2). This discovery held strong in comprehensive sensitivity analyses. We did not find a clear link between other plasma lipids and cancer. Our research results powerfully demonstrated the role of plasma lipids in cancer risk.

## 1. Background

As of now, cancer conditions are deemed the most severe, owing to protracted treatment durations and costly methods. In spite of this, survival percentages are lower, especially in less developed nations that constitute the bulk of the global population. This is primarily due to constraints in early cancer detection. Once cancer metastasizes throughout the body, it becomes challenging to control and frequently leads to fatality. As per the World Health Organization, there has been a rising trajectory in the worldwide cancer burden, with an estimated 18.1 million fresh cancer diagnoses in 2018. Furthermore, cancer is globally recognized as the 2nd most common cause of mortality, responsible for nearly 9.6 million fatalities in 2018 alone.^[1–4]^

Within blood and tissues, both dietary and internally produced fatty acids exist either freely or as components of intricate lipid structures, cumulatively constituting the lipidome of the corresponding tissue. The plasma lipid reservoir originates from various tissues and incorporates both alterable factors (such as routine diet, physical exercise, and fasting condition) and unalterable elements (like cancer).^[5]^ Consequently, plasma lipid molecular species are frequently utilized for clinical purposes in predicting the risk of various diseases, such as type 2 diabetes,^[6]^ gestational diabetes,^[7]^ nonalcoholic fatty liver disease,^[8]^ and cancer.^[9–11]^ Particularly in recent years, an increasing body of evidence underscores the intimate link between plasma lipid species and the progression of cancers. Plasma phospholipids and sphingolipids exhibit the most significant changes during the progression of ovarian cancer.^[12]^ Meanwhile, in patients with breast cancer, renal cancer, and prostate cancer, a combination of potential biomarkers for screening includes plasma cholesteryl ester 16:0, lysophosphatidylcholine 18:2, ceramide 42:1, phosphatidylcholine 36:2, sphingomyelin 32:1, sphingomyelin 41:1, and phosphatidylcholine 36:3.^[13]^ However, executing randomized controlled trials can be a daunting task, given the logistical complexities and financial implications. The impact of plasma lipid species on cancers has not been extensively studied in sufficiently powered trials. Furthermore, deriving causal relationships from conventional observational studies can be fraught with difficulties due to the potential for residual confounding and reverse causality.

Mendelian randomization (MR) offers a unique approach to ascertain causal evidence. MR leverages single nucleotide polymorphisms (SNPs) pinpointed by genome-wide association studies (GWASs) as genetic instruments to assess the influence of an exposure (for instance, plasma phosphatidylethanolamine (18:0_20:4)) on the risk of an outcome (such as pancreatic cancer). GWASs have effectively detected numerous genetic variants implicated in plasma lipid species.^[14]^ Crucially, given that these genetic variants are randomly assigned at conception, MR investigations are considerably less prone to reverse causation and confounding compared to conventional observational studies.^[15]^

The aim of this research is to ascertain the correlation between the genetically predicted plasma lipidome and the genetically predicted incidence of cancer. In a comprehensive GWAS and bidirectional MR analysis, we identified significant associations between 20 plasma lipid species and the risk of 5 types of cancer, including pancreatic cancer, lung cancer, breast cancer (estrogen receptor [ER]+), endometrial cancer (endometrioid histology), and colorectal cancer. Furthermore, we employed colocalization analysis to predict the likelihood of shared causal variants between plasma lipid species and cancers, thereby reinforcing the evidence of their association.

## 2. Methods

### 2.1. Study design

The study design is comprehensively illustrated in Figure 1. We employed a 2-sample MR approach, utilizing aggregated genetic associations from various GWAS, to investigate the intricate causal relationship between 179 plasma lipid species and 15 different cancers. These 15 different cancers include pancreatic cancer, lung cancer, colorectal cancer, breast cancer, endometrial cancer, bladder cancer, cervical cancer, esophageal cancer, gastric cancer, kidney cancer, liver cancer, melanoma, ovarian cancer, prostate cancer, and thyroid cancer. Using a unified MR framework (Fig. 1) – including instrumental variable selection and multiple statistical models – we systematically tested all possible lipid–cancer pairings (a total of 179 × 15 associations), thereby establishing genetic connections between the 2 domains. This design ensures that the identified associations are comprehensive and data-driven, rather than subjectively selected. The cohorts for both exposure and outcome were limited to individuals of European descent to minimize bias due to population stratification. The data utilized in this study are open to the public, originating from studies with appropriate participant consent and ethical clearance. Consequently, the current study did not require additional approval from an institutional review board.

**Figure 1.**
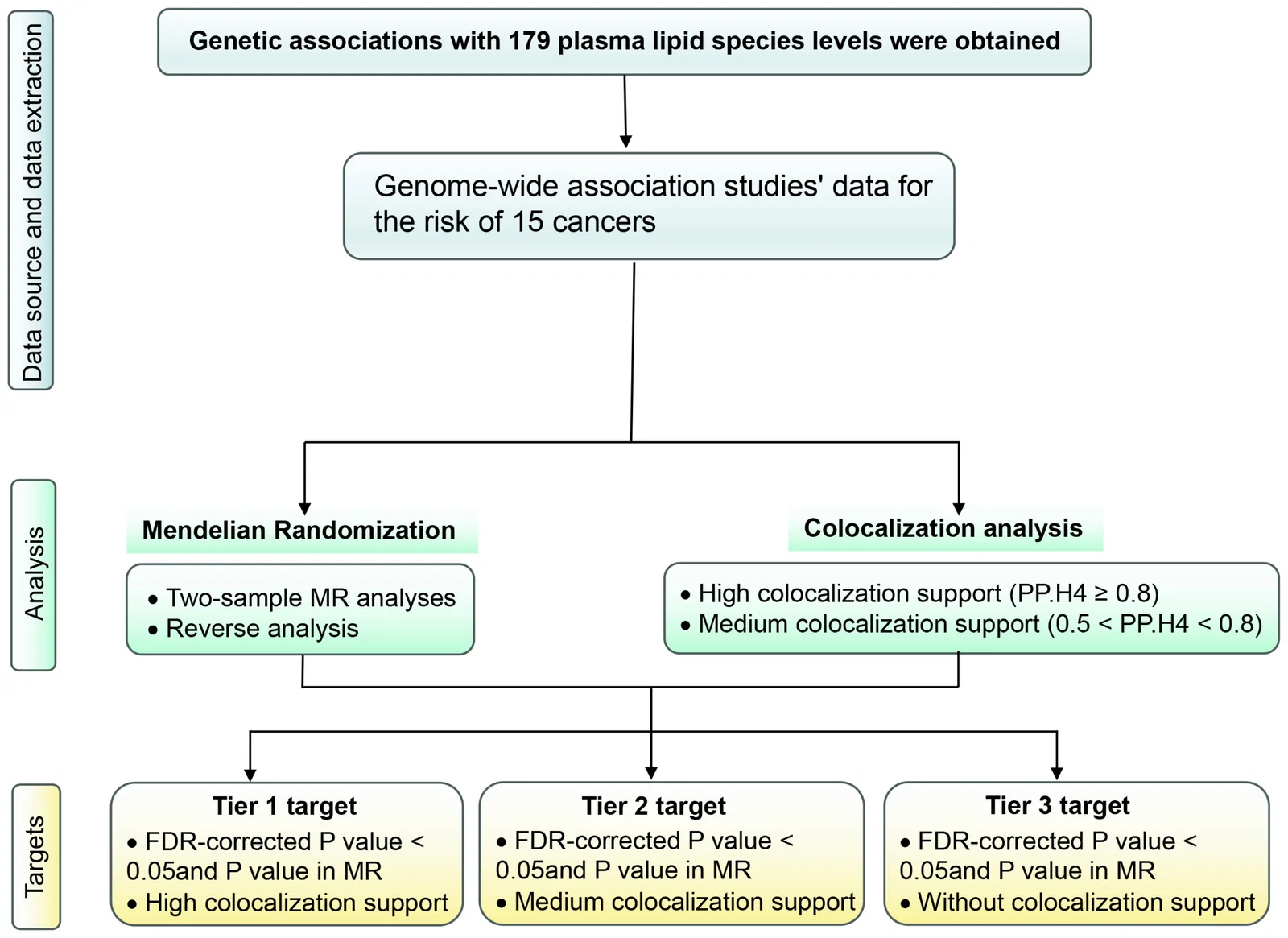
Study design. FDR = false discovery rate, MR = Mendelian randomization, PP.H4 = posterior probability of hypothesis 4.

In summary, this study followed the standard paradigm of 2-sample bidirectional MR analysis. The core steps included: data preparation – independent acquisition of GWAS summary statistics for 179 lipid species and 15 cancer types; instrumental variable selection – identifying significant and independent SNPs for each lipid trait; genetic harmonization – coordinating and clustering SNPs across datasets; bidirectional MR analysis – evaluating the causal effects of lipids on cancer risk and vice versa; and robustness validation – including sensitivity analyses, pleiotropy tests, and colocalization analyses. This sequence of steps forms a complete and logically rigorous analytical pipeline, ensuring the reliability of our findings.

### 2.2. Data source of 179 plasma lipid species

The GWAS data involved 179 plasma lipids across 13 lipid classes, derived from 7174 non-blood-related Finnish individuals from the GeneRISK cohort. The subjects in question were subjected to comprehensive genomic typing, and their plasma lipid species were measured with great precision. Utilizing shotgun lipidomics, Ottensmann L identified 179 species of lipids that fall under 13 lipid classes spanning 4 primary lipid categories: glycerophospholipids, glycerolipids, sphingolipids, and sterols.^[16]^ Lipid molecules were discerned at both the species and subspecies levels. The nomenclature for lipid species follows this format: class name :;. Annotations for lipid subspecies encompass details about their acyl constituents and, when accessible, information about their sn-position. We have provided all the data sources of 179 plasma lipid species, along with the download websites (Table S1, Supplemental Digital Content 1).

### 2.3. Data source of 15 types of cancer

We conducted a comprehensive search for published GWASs that assessed individuals of European descent, utilizing resources such as the GWAS Catalog and PubMed. The most recent search was carried out in January 2024. We focused on the outcomes of risk for 17 types of cancer: pancreatic cancer, lung cancer, colorectal cancer, breast cancer, endometrial cancer, bladder cancer, cervical cancer, esophagus cancer, gastric cancer, kidney cancer, liver cancer, melanoma, ovarian cancer, prostate cancer, thyroid cancer, corpus uteri cancer, and oral cavity pharyngeal cancer.^[17–24]^ Taking into account factors such as a small sample size, insufficient quality control of the samples, and low heritability of the traits, we excluded corpus uteri cancer and oral cavity pharyngeal cancer from our study. We only considered the remaining 15 types of cancer. In addition, considering the biological behavior of cancer, treatment responses, and prognostic differences, we categorized breast cancer, kidney cancer, and endometrial cancer in more detail. For example, breast cancer was classified based on the status of the ER (positive or negative), kidney cancer was classified based on gender, and endometrial cancer was classified based on histological characteristics (endometrioid and non-endometrioid). Table S2, Supplemental Digital Content 2 describes the data sources used for these disease endpoints, including the source of the database, the number of cases, summary statistics, and related information.

### 2.4. Filtering of SNPs

The careful choice of suitable SNPs is crucial for the successful implementation of MR analysis. The fundamental premise of MR requires that all SNPs predict exposure independently and significantly at the genome-wide level of significance. Applying a genome-wide significance threshold (*P* < 5 × 10^−8^) would have led to the omission of a significant number of SNPs. As a result, we opted for a more lenient but still statistically significant threshold of 5 × 10^−6^, as suggested by prior research, to include the majority of SNPs associated with the 179 plasma lipid species.^[25]^ We imposed constraints of *r*^2^ < 0.001 and kb = 10,000 to alleviate potential linkage disequilibrium (LD). Palindromic SNPs underwent rigorous examination for precise effect allele alignment, with any ambiguous instances being omitted. Additionally, SNPs that were weakly substantiated with *F* values (*F* = *r*^2^ × (N − 2)/ (1 − *r*^2^)) falling below 10 were discarded to bolster the robustness of the association between the instrumental variables and exposure. These stringent screening parameters ensured the credibility of the results in our study.

### 2.5. Two-sample MR

A thorough 2-sample MR analysis was conducted to evaluate the causal relationships between 179 plasma lipid species and the risk of 15 different types of cancers (as depicted in Fig. 2). Estimates for individual SNPs were derived using instrumental variable ratios. Presuming the validity of instruments devoid of pleiotropy, we carried out a range of analyses including inverse variance weighted (IVW), MR-Egger, weighted median, weighted mode, and simple mode to examine the causal link between exposure factors and outcomes.^[26]^ Moreover, a comprehensive sensitivity analysis was conducted using a variety of methods to confirm the reliability of the results. This included the investigation of heterogeneity, evaluation of horizontal pleiotropy, examination of funnel plots, and execution of a leave-one-out analysis. The principal MR analysis, IVW, was chosen for those instances with 2 or more SNPs. This was done to assess the potential causal impact of 179 plasma lipid species on the risk associated with 15 distinct types of cancers. Variability in individual causal effects was evaluated using *Q*-statistics, with *P* values <.05 signifying the existence of heterogeneity. To tackle horizontal pleiotropy, both MR-Egger regression and the MR Pleiotropy Residual Sum and Outlier test were utilized for rectification purposes. Considering the inherent risk of false positives that can occur from concurrently processing and comparing multiple datasets, we utilized the false discovery rate (FDR) correction test. This was done to assess the robustness of the causal relationship between the exposure and outcome variables. We opted not to use Bonferroni correction because, compared to it, FDR correction is more lenient. However, it allows for the discovery of more true differences while controlling the rate of false discoveries. On the other hand, Bonferroni correction, while being the simplest method of correction, is also the strictest and significantly increases the rejection rate of type I errors.^[27]^ An FDR-corrected *P* value <.05 is considered significant.

**Figure 2.**
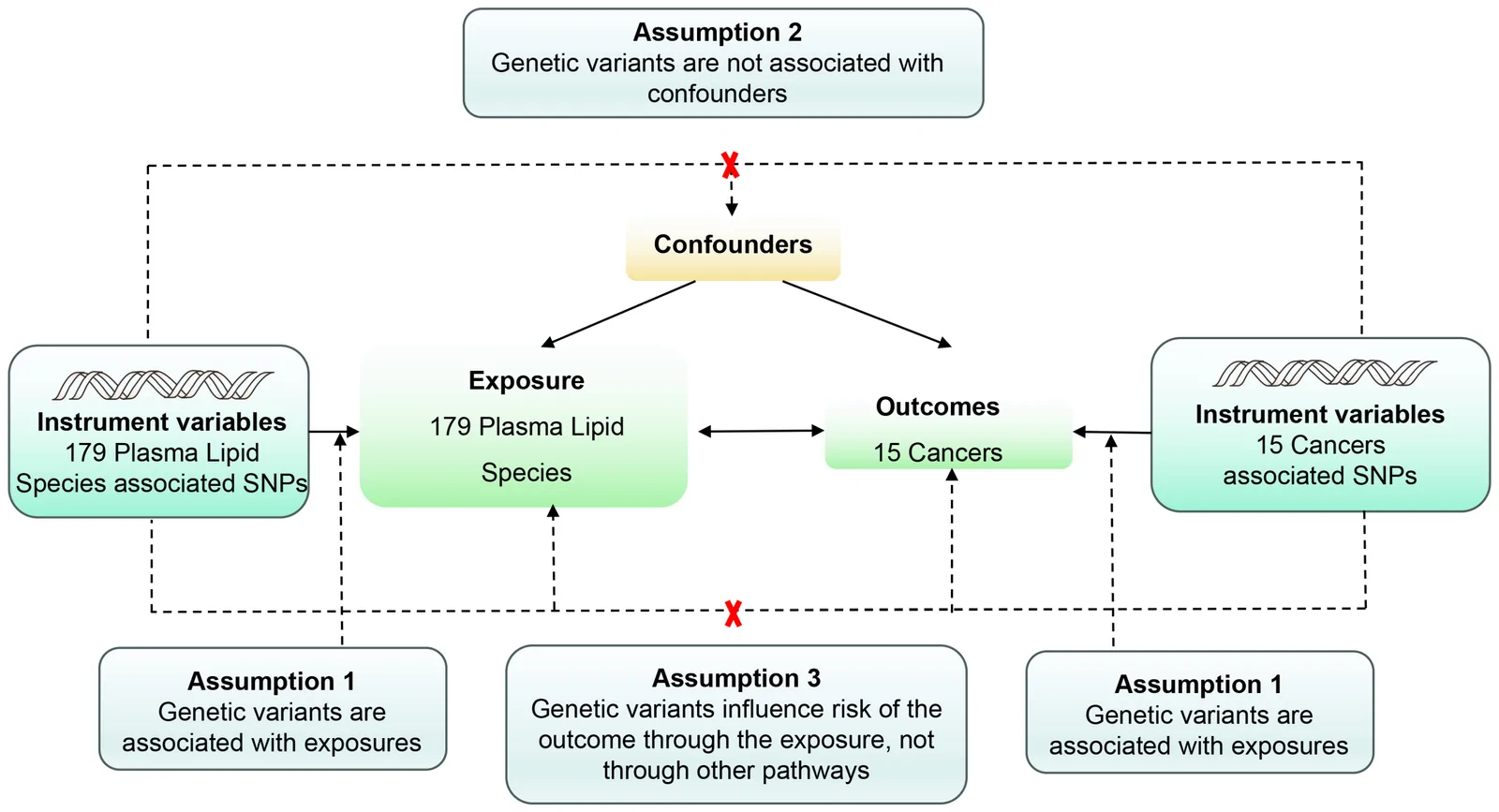
Assumptions of a Mendelian randomization analysis for 179 plasma lipid species and the risk of 15 different types of cancers. Dashed lines represent potential pleiotropic or direct causal relationships between variables that could infringe upon the assumptions of Mendelian randomization. SNPs = single nucleotide polymorphisms.

### 2.6. Colocalization

The colocalization analysis, an integral part of our study, was performed utilizing the coloc R package (R Foundation for Statistical Computing).^[28]^ Colocalization analysis is a method used to study the association between genetic variations and phenotypes. A positive result in colocalization analysis means that a specific genetic variation shares the same genetic location with signals associated with both exposure and outcome. It is particularly useful for testing whether the signals observed in the genetic correlation studies of 179 plasma lipid species and the risk of 15 different types of cancers originate from the same genetic variation. Similarly, colocalization analysis can corroborate the evidence of causal relationships in MR by reducing the possibility of pleiotropy and enhancing the association that genetic variations directly affect exposure. Utilizing a Bayesian methodology, the study examined 5 mutually exclusive hypotheses for the locus: absence of association with both traits; exclusive association with the 1st trait; exclusive association with the 2nd trait; both traits linked to unique causal variants; and both traits influenced by the same causal variant. Each hypothesis test (*H*0, *H*1, *H*2, *H*3, and *H*4) was assigned posterior probabilities. It is worth mentioning that the a priori probabilities for the SNP’s linkage with only trait 1 (*p*1) and solely trait 2 (*p*2) were established at 1 × 10^−4^ each. Concurrently, the likelihood of the SNP associating with both traits (*p*3) was fixed at 1 × 10^−5^. The strength of colocalization evidence was characterized by posterior probabilities. A value of posterior probability of hypothesis 4 (PP.H4) ≥ 0.8 was considered substantial evidence of colocalization, while an intermediate colocalization indication was represented by 0.5 < PP.H4 < 0.8.

A total of 179 plasma lipid species-outcome associations, with an FDR-corrected *P* value <.05 in MR, were subsequently segregated into 3 distinct categories. The plasma lipid species that exhibited high-support evidence of colocalization (PP.H4 ≥ 0.8) were classified as tier 1 targets. Those displaying medium-support evidence of colocalization (0.5 < PP.H4 < 0.8) were grouped as tier 2 targets. The remaining lipid species, which did not show significant colocalization evidence, were categorized as tier 3 targets. This detailed stratification provides a comprehensive understanding of the colocalization strength and aids in the subsequent ranking of the 179 plasma lipid species for future exploration.

## 3. Results

### 3.1. Main analyses

We included a total of 179 plasma lipid species in subsequent MR analyses. We conducted quality control for SNPs associated with plasma lipid species, using a locus-wide significance threshold of *P* < 5 × 10^−6^. Subsequently, we applied harmonization and clumping procedures to remove palindrome SNPs and reduce the impact of linkage LD. In addition, after determining the number of instrumental variables (IVs) related to 179 plasma lipid species associated with 15 types of cancer, we performed FDR correction to ensure the robustness of the causal relationship between exposure and outcome variables. Relevant data for SNPs meeting the criteria were provided in Table S3, Supplemental Digital Content 3. Importantly, all IVs exhibited *F* statistics exceeding the threshold of 10, indicating the absence of weak instrument bias.

We analyzed the causal relationship between 179 plasma lipid species and the risk of 15 types of cancer. Our investigation confirmed that only 20 plasma lipid species exhibit a causal relationship with the risk of 5 types of cancer (Fig. 3, Table S4, Supplemental Digital Content 4). We conducted genetic prediction using the IVW method and further validated it using odds ratios (ORs) and 95% confidence intervals (CIs). When plasma levels of phosphatidylethanolamine (18:0_20:4) were lower, the risk of pancreatic cancer increased (OR = 0.795, 95% CI: 0.705–0.896, FDR-corrected *P* value = .030). Elevated plasma levels of phosphatidylcholine (20:4_0:0) (OR = 1.109, 95% CI: 1.046–1.176, FDR-corrected *P* value = .035) and phosphatidylcholine (O-18:2_20:4) (OR = 1.197, 95% CI: 1.081–1.325, FDR-corrected *P* value = .035) were associated with an increased risk of lung cancer. Conversely, lower levels of phosphatidylcholine (18:1_20:2) were linked to a decreased risk of lung cancer (OR = 0.896, 95% CI: 0.842–0.954, FDR-corrected *P* value = .035). In breast cancer (ER+) and breast cancer (ER−), lower plasma levels of phosphatidylcholine (O-18:0_16:1) were associated with an increased risk of breast cancer (ER+) (OR = 0.905, 95% CI: 0.859–0.953, FDR-corrected *P* value = .027), while they were not related to breast cancer (ER−). In both endometrial cancer (endometrioid histology) and endometrial cancer (non-endometrioid histology), higher plasma levels of phosphatidylcholine (O-16:0_20:3) were associated with a significantly increased risk of developing endometrial cancer (endometrioid histology) (OR = 1.262, 95% CI: 1.137–1.401, FDR-corrected *P* value = .002), while there was no association with endometrial cancer (non-endometrioid histology).

**Figure 3.**
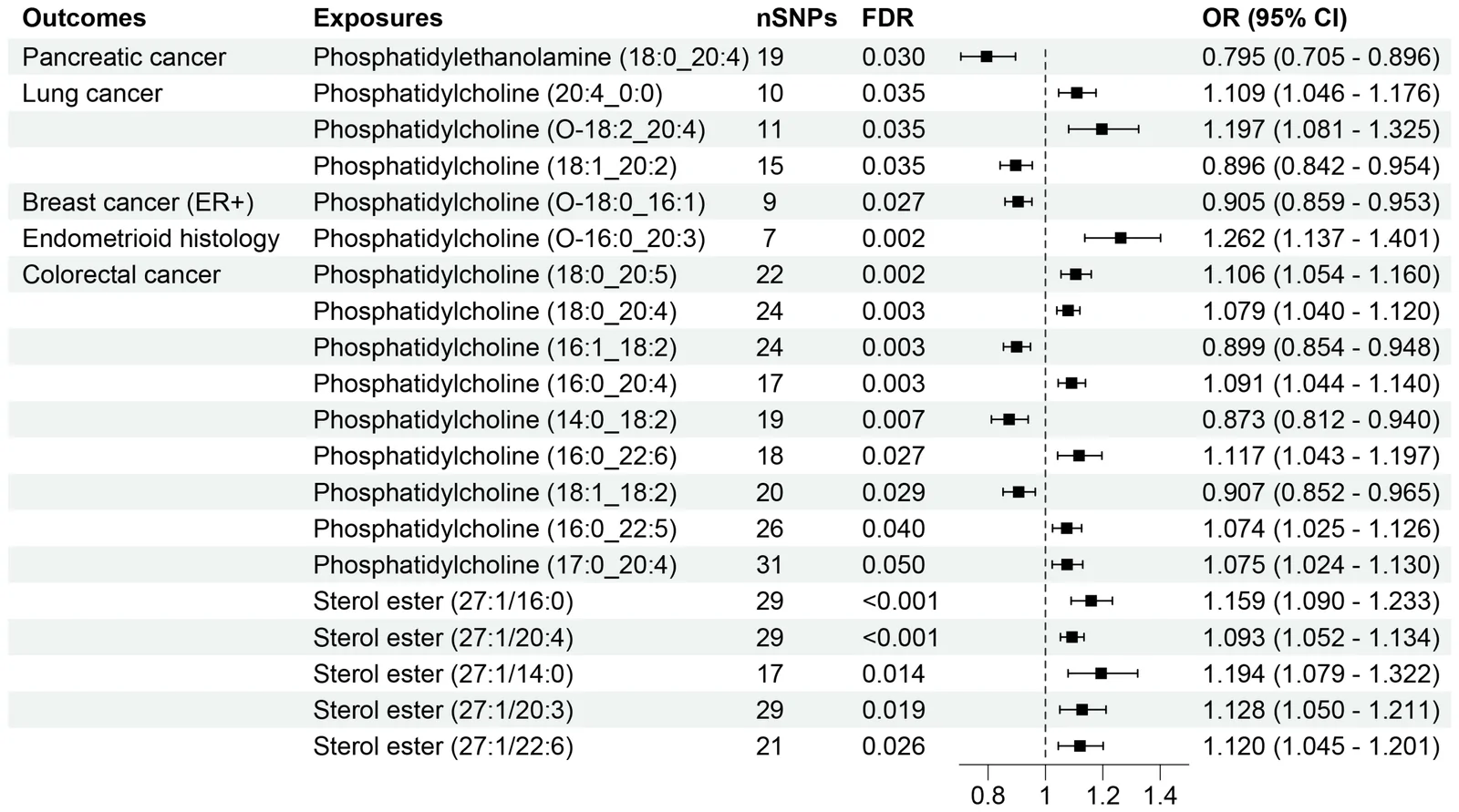
Forest of 20 plasma lipid species with risk of 5 types of cancer. CI = confidence intervals, ER = estrogen receptor, FDR = false discovery rate, OR = odds ratios, SNPs = single nucleotide polymorphisms.

Notably, the causal relationship between plasma lipids and the risk of colorectal cancer was the strongest. Furthermore, compared to other cancers, not only were 14 plasma lipid species associated with colorectal cancer risk, but the FDR-corrected *P* values were also more significant. Elevated levels of 11 plasma lipid species were associated with an increased risk of colorectal cancer. These included phosphatidylcholine (18:0_20:5), with an OR of 1.106 (95% CI: 1.054–1.160, FDR-corrected *P* = .002), and phosphatidylcholine (18:0_20:4), with an OR of 1.079 (95% CI: 1.040–1.120, FDR-corrected *P* = .003). Phosphatidylcholine (16:0_20:4) and (16:0_22:6) also demonstrated elevated ORs of 1.091 (95% CI: 1.044–1.140, *P* = .003) and 1.117 (95% CI: 1.043–1.197, *P* = .027), respectively. Additionally, phosphatidylcholine (16:0_22:5) and (17:0_20:4) were associated with ORs of 1.074 (95% CI: 1.025–1.126, *P* = .040) and 1.075 (95% CI: 1.024–1.130, *P* = .050). Sterol esters also exhibited notable associations. Sterol ester (27:1/16:0) had an OR of 1.159 (95% CI: 1.090–1.233, *P* < .001), while sterol ester (27:1/20:4) showed an OR of 1.093 (95% CI: 1.052–1.134, *P* < .001). Other significant findings included sterol ester (27:1/14:0) with an OR of 1.194 (95% CI: 1.079–1.322, *P* = .014), sterol ester (27:1/20:3) with an OR of 1.128 (95% CI: 1.050–1.211, *P* = .019), and sterol ester (27:1/22:6) with an OR of 1.120 (95% CI: 1.045–1.201, *P* = .026). Increased levels of these 11 plasma lipids were associated with a higher risk of colorectal cancer, as similarly confirmed by MR-Egger and weighted median analyses. In contrast, Wald analysis indicated that lower levels of phosphatidylcholine (16:1_18:2) (OR = 0.899, 95% CI: 0.854–0.948, FDR-corrected *P* value = .003), phosphatidylcholine (14:0_18:2) (OR = 0.873, 95% CI: 0.812–0.940, FDR-corrected *P* value = .007), and phosphatidylcholine (18:1_18:2) (OR = 0.907, 95% CI: 0.852–0.965, FDR-corrected *P* value = .029) were associated with an increased risk of colorectal cancer.

Further assessments, including MR-Egger regression, MR Pleiotropy Residual Sum and Outlier, and Cochrane *Q* test, confirmed the absence of horizontal pleiotropy, outliers, or significant heterogeneity in the selected SNPs (*P* > .05) (Table S4, Supplemental Digital Content 4). Additionally, in Figure S1, S2 and S3, Supplemental Digital Content 5, we provided scatter plots of the genetic association of 6 plasma lipid species on the risk of 4 types of cancer, along with leave-one-out sensitivity analyses and forest plots. In Figure S4, S5 and S6, Supplemental Digital Content 6, we provided scatter plots of the genetic association of 14 plasma lipid species on the risk of colorectal cancer, along with leave-one-out sensitivity analyses and forest plots. These visualizations help evaluate whether the observed associations are likely to be causal or influenced by confounding factors.

### 3.2. Colocalization

To support the evidence of causality in MR studies, we conducted a rigorous colocalization analysis on 20 plasma lipid species with the risk of 5 types of cancer. The objective of this study was to ascertain if the observed correlations between 20 plasma lipid species and the risk of 5 different cancers originated from identical genetic determinants. The results showed strong evidence of high-support colocalization between 11 plasma lipid species (levels of phosphatidylcholine [18:0_20:5], phosphatidylcholine [16:1_18:2], phosphatidylcholine [16:0_20:4], phosphatidylcholine [14:0_18:2], phosphatidylcholine [16:0_22:6], phosphatidylcholine [18:1_18:2], phosphatidylcholine [16:0_22:5], phosphatidylcholine [17:0_20:4], sterol ester [27:1/16:0], sterol ester [27:1/20:4], and sterol ester [27:1/22:6]) and colorectal cancer, and these were designated as tier 1 targets (Fig. S7, Supplemental Digital Content 7, Table S5, Supplemental Digital Content 8). In addition, it was demonstrated that there was no colocalization support between phosphatidylcholine (18:0_20:4) levels, sterol ester (27:1/14:0) levels, and sterol ester (27:1/20:3) levels and colorectal cancer, and these were designated as tier 3 targets (Fig. S8, Supplemental Digital Content 9, Table S5, Supplemental Digital Content 8). Notably, compared to colorectal cancer, plasma lipids were without colocalization support with the other 4 types of cancers, and thus, they were all defined as tier 3 targets (Fig. S8, Supplemental Digital Content 9, Table S5, Supplemental Digital Content 8). These plasma lipids were pinpointed as tier 1 targets, signifying a sturdy and well-substantiated association. In contrast, the plasma lipids-outcome were appropriately classified as tier 3 targets, indicating their limited evidence of colocalization.

### 3.3. Reverse analysis

Our study unveiled robust causal links between 20 plasma lipid species and 5 cancer outcomes, as evidenced by forward MR analyses. In a corresponding reverse MR analysis, we delved into the genetic interplay between these 5 types of cancer and the 20 plasma lipid species. We also conducted quality control on SNPs associated with the 5 types of cancer, adopting a genome-wide significance threshold of *P* < 5 × 10^−6^. Through harmonization and clustering procedures, we eliminated palindromic SNPs to reduce the impact of LD. Moreover, we ensured that the *F* statistics of all IVs exceeded a threshold of 10. The relevant data for the SNPs that met the criteria can be found in Table S6, Supplemental Digital Content 10. The results of the reverse analysis indicated that the genetic prediction of an increased risk of colorectal cancer was associated with higher plasma levels of phosphatidylcholine (18:1_18:2) (β = 0.063, 95% CI: 0.011–0.115, *P* value = .017) (Fig. 4). Notably, this situation was not observed in other cancers and plasma lipids. Crucially, throughout the examination, we found no signs of horizontal pleiotropy (*P* > .05), which underscores the solidity and dependability of our results (refer to Tables S7, Supplemental Digital Content 11).

**Figure 4.**
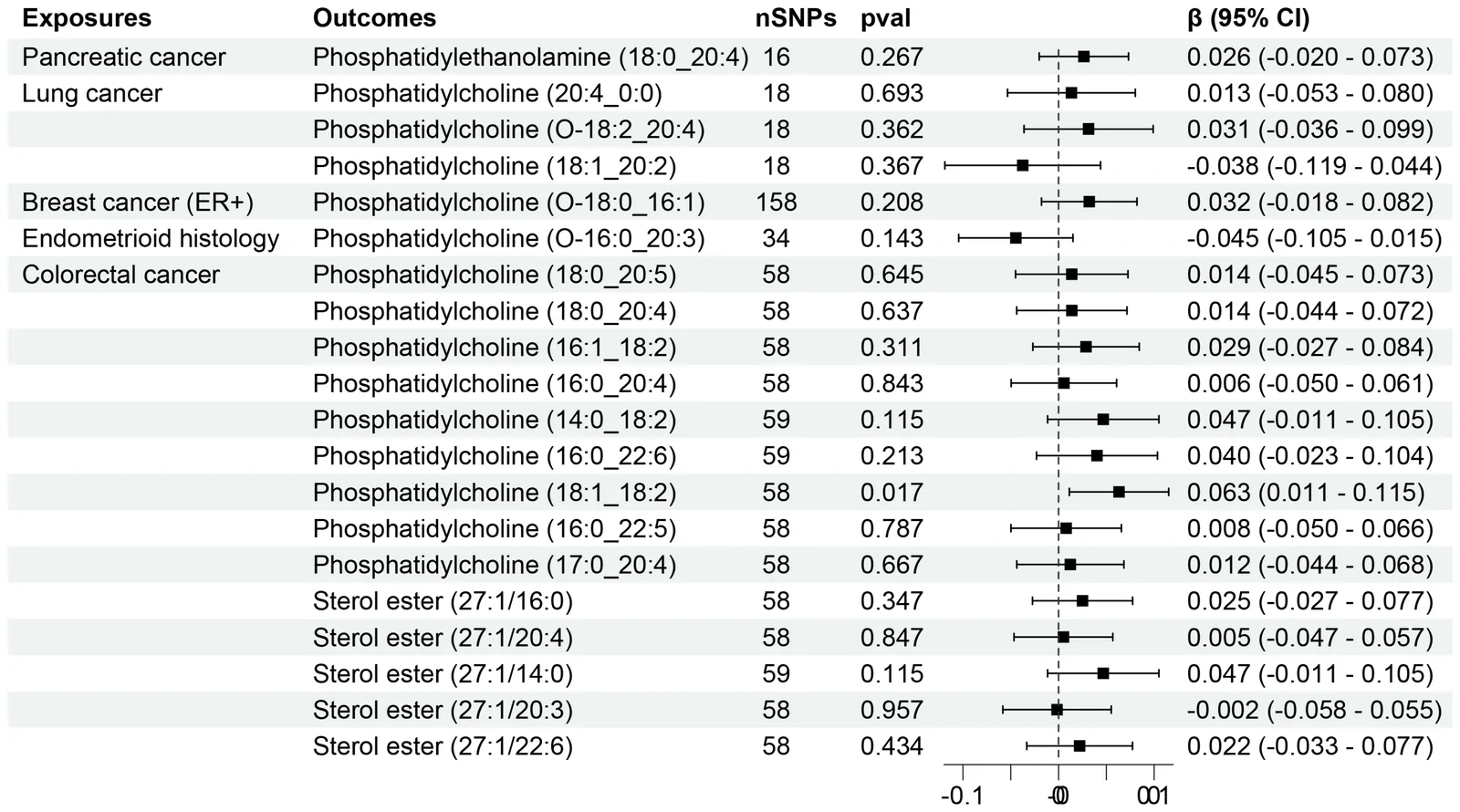
Forest for association of genetically predicted risk of 5 types of cancer with 20 plasma lipid species. CI = confidence intervals, ER = estrogen receptor, SNPs = single nucleotide polymorphisms.

## 4. Discussion

In this MR study on 179 plasma lipid species and the risk of 15 types of cancer, we found that the genetically predicted levels of 20 plasma lipid species are closely related to the genetically predicted risk of 5 types of cancer. We did not find a clear link between other plasma lipids and cancer. It is noteworthy that this study provided several pieces of information worth further investigation. First, the study involved a wide variety of plasma lipids, mainly including sterol ester, ceramide, cholesterol, phosphatidylcholine, phosphatidylethanolamine, phosphatidylinositol, sphingomyelin, triacylglycerol, and diacylglycerol. However, the genetically predicted plasma phosphatidylcholine levels were most closely related to the risk of 5 types of cancer. Second, the study confirmed that the genetically predicted levels of 5 plasma sterol esters were closely related to the risk of colorectal cancer. Interestingly, all 5 plasma sterol ester levels were positively correlated with the risk of colorectal cancer; i.e., an increase in the levels of all 5 plasma sterol esters was associated with an increased risk of colorectal cancer. Third, in this MR study, a clear link was found between 20 plasma lipid species and 5 types of cancer. However, 14 of these plasma lipid species were closely related to the risk of colorectal cancer. The colocalization analysis also highlighted colorectal cancer. Fourth, breast cancer was classified according to the status of ER (ER+ or ER−), and endometrial cancer was classified according to histological characteristics (endometrioid histology and non-endometrioid histology). Plasma phosphatidylcholine (O-18:0_16:1) and phosphatidylcholine (O-16:0_20:3) were closely related to breast cancer (ER+) and endometrial cancer (endometrioid histology), but not to breast cancer (ER−) and endometrial cancer (non-endometrioid histology).

Phosphatidylcholine, phosphatidylethanolamine, and phosphatidylserine, all of which are phospholipids, play a pivotal role in the formation of biological membranes. They contribute to the fluidity of the lipid bilayer, which is essential for preserving the selective permeability and structural robustness of cells.^[29]^ It is noteworthy that the hydrolysis of phosphatidylcholine leads to the production of lipid mediators. These mediators facilitate intercellular communication that promotes the survival and proliferation of cancer cells. They also modulate immune responses, ultimately resulting in therapy resistance. Moreover, precursors or degradation products of phosphatidylcholine often accumulate in cancer cells. These components provide the necessary building blocks to fuel their synthetic metabolic phenotype and promote intracellular processes that lead to drug resistance.^[30]^ Therefore, many studies often use the levels of phosphatidylcholine in plasma and its degradation product, lysophosphatidylcholine, as general indicators of the severity of cancer.^[31,32]^ Collectively, these mechanisms underscore phosphatidylcholine’s central role not only in cellular integrity but also in cancer progression and treatment resistance.

Building on these mechanistic insights, clinical evidence further corroborates the significant association between phosphatidylcholine profiles and cancer development. In a study related to the staging of colorectal cancer and associated plasma metabolites, it was found that, compared to stage I patients, stage IV patients had lower levels of phosphatidylcholine (acyl-alkyl) C40:1, phosphatidylcholine (diacyl) C34:4, and lysophosphatidylcholines (acyl) C16:0 and C17:0 in their plasma. Conversely, the level of phosphatidylcholine (diacyl) C32:0 in the plasma was higher in stage IV patients than in stage I patients.^[33]^ A study on the lipid composition of small extracellular vesicles derived from lung cancer serum found that the content of phosphatidylcholine in the vesicles of lung cancer patients decreased.^[34]^ In the comparison between cervical cancer patients and a healthy control group, 31 types of plasma lipids showed abnormal changes in cervical cancer. Among them, phosphatidylcholine was the main lipid category of these 31 lipids and was considered a potential method for detecting the progression of cervical cancer.^[35]^ These clinical data indirectly confirmed the importance of phosphatidylcholine and also explained why our key information suggested that phosphatidylcholine was the lipid in plasma most closely related to cancer. However, due to the small sample size of traditional experiments, the involvement of a single lipid category, and the limited scope of research, the relationship between many different types of plasma phosphatidylcholine and various cancers remained unclear. Our research complemented these clinical experiments, providing a more rigorous and comprehensive explanation from a genetic level and offering ideas and directions for subsequent research.

In the human body, cholesterol ester was the most significant type of sterol ester, occupying a considerable proportion. It was the absolute focus of researchers’ studies. Therefore, all 15 types of sterol esters mentioned in this paper were cholesterol esters. Among them, high levels of 5 types of cholesterol esters were closely associated with an increased risk of colorectal cancer. Cholesterol ester was the esterified product of cholesterol, serving as a storage and transport form of cholesterol. Excess cholesterol was converted into cholesterol esters by cholesterol esterification enzyme and stored in lipid droplets, which were then released into the blood. Therefore, the level of cholesterol esters in the blood often reflected the body’s cholesterol homeostasis.^[36]^ Cholesterol and cholesterol esters were demonstrated to play a role in regulating signaling pathways for the growth, proliferation, and metastasis of cancer cells. Recent studies suggested that cholesterol metabolism could produce tumor promoters, such as cholesterol esters and 27-hydroxycholesterol.^[37]^ Cholesterol esterification promoted the development of colorectal cancer in a SOAT1-dependent manner.^[38]^ Cholesterol metabolism disorder was considered a hallmark of colorectal cancer. Targeting the cholesterol-retinoic acid receptor-related orphan receptor α/γ axis could inhibit the progression of colorectal cancer.^[39]^ Cholesterol-lowering drugs were used to reduce the metastatic and invasive nature of colorectal cancer to influence outcomes.^[40–42]^ Thus, the body of evidence firmly establishes cholesterol and its esters as key contributors to colorectal cancer pathogenesis.

While these mechanistic insights are critical, translating them into practical diagnostic applications requires a clearer understanding of the specific roles played by circulating plasma cholesterol esters. These studies went to great lengths to explore the molecular mechanisms of cholesterol and cholesterol esters in relation to colorectal cancer. However, the rise in plasma cholesterol ester levels was often closely related to the overproduction of cholesterol in tissues. In the diagnosis and detection of colorectal cancer, the convenient predictive role of plasma cholesterol esters was irreplaceable. It was worth noting that there were always 2 key issues overlooked in colorectal cancer and plasma cholesterol esters. Which plasma cholesterol esters had potential effects on colorectal cancer? Were all plasma cholesterol esters risk factors for colorectal cancer? Our research provided answers: out of the 15 most important cholesterol esters in plasma, only 5 are closely related to the risk of colorectal cancer, and all are related to an increased risk of colorectal cancer at high levels. Our research also raised a question; surprisingly, plasma cholesterol levels are not related to the risk of colorectal cancer or other cancer risks.

An increasing number of studies had emphasized that both the abnormal lipid metabolism in cancer cells and the changes in plasma lipid levels were related to colorectal cancer. In colorectal cancer, the accumulation of lipid droplets could support the chemotherapy resistance of cancer cells,^[43]^ promote cancer cells to acquire stem cell-like characteristics,^[44]^ and accelerate the progression of cancer.^[45]^ Metabolomic analysis of plasma in patients with colorectal cancer revealed that several lipid subclasses, including phosphatidylcholine, fatty acids, phosphatidylethanolamine, ceramide, and sterol esters, were upregulated during tumor progression.^[46]^ In another study on plasma in colorectal cancer, plasma lysophospholipid levels could potentially be evaluated as biomarkers related to colorectal cancer. Compared with the control group, the levels of 18:1-lysophosphatidylcholine or 18:2-lysophosphatidylcholine in the plasma of patients with colorectal cancer were significantly reduced.^[47]^ Although these clinical studies were not comprehensive, they were sufficient to explain the key role of plasma lipids in colorectal cancer. In our research, we also demonstrated that colorectal cancer, whether in MR analysis or colocalization analysis, had a great correlation with plasma lipids and was absolutely the main character. It was noteworthy that there were studies showing a close correlation between the levels of total cholesterol and triglycerides in the plasma of patients with colorectal cancer and the tumor-node-metastasis staging.^[48]^ Another study also showed a positive correlation between increased plasma triglyceride levels and increased total cholesterol levels and the incidence of colorectal cancer.^[49]^ These collective findings firmly establish plasma lipids as key players in colorectal cancer, a central conclusion strongly supported by our genetic evidence.

Despite this overarching consensus, our genetic approach revealed nuanced and sometimes counterintuitive relationships that merit further discussion. In our research, the genetically predicted levels of cholesterol and triglycerides did not have a significant correlation with the genetically predicted risk of colorectal cancer, which contradicted our research. We speculated that this might be due to different experimental methods, different sample sizes, the influence of confounding factors, and even inconsistencies in the assessment of cancer subsites, which required further exploration.

Another noteworthy phenomenon in this study was that specific phosphatidylcholines (O-18:0_16:1 and O-16:0_20:3) are notably associated with breast cancer (ER+) and endometrial cancer (endometrioid histology), but not with ER− breast cancer and non-endometrioid endometrial cancer. Regrettably, we had not found any relevant studies to provide an explanation for this phenomenon. However, our research conclusions provided a new direction for future researchers: there might have been a potential connection between the biological characteristics of breast cancer and endometrial cancer and metabolic status.

Our research showcased a number of advantages and constraints. By utilizing an MR approach, we minimized the risk of confounding that was common in observational investigations. Moreover, by consolidating aggregated data from various cohorts, we substantially amplified the statistical robustness and diminished stochastic errors. Nonetheless, in spite of the generous sample volumes, certain genetic tools associated with exposures and outcomes were plagued by imprecise phenotype characterizations and inconsistent statistical potency. Within the framework of plasma lipids and various types of cancer, the adoption of more accurate phenotype classifications and expanded sample volumes proved to be advantageous. Furthermore, the employment of summarized data posed difficulties, as it prevented categorization based on elements such as age, dietary habits, usage of lipid-lowering drugs, or concurrent health conditions. The utilization of summary-level data hindered our capacity to pinpoint individuals with dual or multiple combinations, potentially leading to bias. We noted a degree of heterogeneity among the instrumental variables. Despite our application of the random-effects IVW method, coupled with the results of sensitivity analyses to underscore the solidity of our conclusions, we advised that biological interpretations be approached with prudence. Despite these limitations, the MR framework provided a robust foundation for inferring causality, and our findings remained largely consistent across sensitivity analyses.

Notwithstanding the methodological constraints, this study presents substantial strengths and offers a comprehensive genetic perspective on the plasma lipid–cancer relationship. Based on our observations, this was the most comprehensive study to date that used the MR method to bidirectionally evaluate the causal effects of 179 plasma lipid species on the risk of 15 types of cancer. The genetic tools for each plasma lipid had been extensively utilized to evaluate correlations with other phenotypes or diseases, thereby enhancing the validity of this study. In order to lessen the potential for population stratification, we confined our evaluation to individuals of European descent. This, however, constrained the applicability of our results to other ethnic groups. The conclusions of our study were validated through a sequence of sensitivity tests, encompassing the examination of heterogeneity, the appraisal of pleiotropy, and leave-one-out substantiation.

## 5. Conclusions

In conclusion, through extensive genome-wide association studies and bidirectional MR analyses of 179 plasma lipid species and the risk of 15 types of cancer, we confirmed a causal association between 20 plasma lipid species and 5 types of cancer. The causal connections identified underscore potential targets for therapeutic intervention. The specific plasma lipid species identified in our study were phosphatidylethanolamine (18:0_20:4), phosphatidylcholine (20:4_0:0), phosphatidylcholine (O-18:2_20:4), phosphatidylcholine (18:1_20:2), phosphatidylcholine (O-18:0_16:1), phosphatidylcholine (O-16:0_20:3), phosphatidylcholine (18:0_20:5), phosphatidylcholine (18:0_20:4), phosphatidylcholine (16:1_18:2), phosphatidylcholine (16:0_20:4), phosphatidylcholine (14:0_18:2), phosphatidylcholine (16:0_22:6), phosphatidylcholine (18:1_18:2), phosphatidylcholine (16:0_22:5), phosphatidylcholine (17:0_20:4), sterol ester (27:1/16:0), sterol ester (27:1/20:4), sterol ester (27:1/14:0), sterol ester (27:1/20:3), and sterol ester (27:1/22:6). Colocalization analysis bolstered these findings, providing substantial proof of shared genetic variants for 11 plasma lipid species associated with the risk of colorectal cancer, all of which were categorized as tier 1. These findings have deepened our understanding of the mechanisms of cancer development driven by plasma lipids, paving the way for targeted interventions and personalized treatment strategies, thereby alleviating the burden of this disease.

## Acknowledgments

The authors thank the participants of all GWAS cohorts included in the present work without whom this effort would not be possible.

## Author contributions

**Conceptualization:** Guangxi Piao, Xinguo Yang.

**Data curation:** Xinguo Yang.

**Formal analysis:** Guangxi Piao.

**Investigation:** Guangxi Piao, Xiaofeng Cui.

**Methodology:** Guangxi Piao, Xinguo Yang.

**Project administration:** Xiaofeng Cui.

**Resources:** Guangxi Piao, Xinguo Yang.

**Software:** Guangxi Piao.

**Validation:** Guangxi Piao.

**Supervision:** Xiaofeng Cui.

**Visualization:** Xiaofeng Cui.






























