# Supplementary material for: Unveiling the association between plasma lipid species and pan-cancers: A bidirectional 2-sample Mendelian randomization study
Source: Medicine (Baltimore). 2026 Sep 25;105(39):e50711. doi: 10.1097/MD.0000000000050711 (PMC13619218; doi:10.1097/MD.0000000000050711)
Supplement: Supplementary file 1 [file medi-105-e50711-s007.docx]

**Supplementary Figure S7.** Regional association plots for the colocalization analysis of 11 plasma lipid species with the risk of colorectal cancer (tier 1 targets, i.e., PP.H4＞0.8). Phosphatidylcholine (18:0_20:5) levels with colorectal cancer(A), phosphatidylcholine (16:1_18:2) levels with colorectal cancer(B), phosphatidylcholine (16:0_20:4) levels with colorectal cancer(C), phosphatidylcholine (14:0_18:2) levels with colorectal cancer(D), phosphatidylcholine (16:0_22:6) levels with colorectal cancer(E), phosphatidylcholine (18:1_18:2) levels with colorectal cancer(F), phosphatidylcholine (16:0_22:5) levels with colorectal cancer(G), phosphatidylcholine (17:0_20:4) levels with colorectal cancer(H), sterol ester (27:1/16:0) levels with colorectal cancer(I), sterol ester (27:1/20:4) levels with colorectal cancer(J), sterol ester (27:1/22:6) levels with colorectal cancer(K). The lead SNP is shown as a purple diamond. SNPs within ± 1000 kb of the lead SNP were included; p12=1e-5, prior probability a SNP is associated with both plasma lipid species and colorectal cancer.
