# Supplementary material for: Unveiling the association between plasma lipid species and pan-cancers: A bidirectional 2-sample Mendelian randomization study
Source: Medicine (Baltimore). 2026 Sep 25;105(39):e50711. doi: 10.1097/MD.0000000000050711 (PMC13619218; doi:10.1097/MD.0000000000050711)
Supplement: Supplementary file 2 [file medi-105-e50711-s005.doc]

**Supplementary Figure S1. Scatter plots of the genetic association of 6 plasma lipid species on the risk of 4 types of cancer.** phosphatidylethanolamine(18:0_20:4) levels with pancreatic cancer(A), phosphatidylcholine(20:4_0:0) levels with lung cancer(B), phosphatidylcholine(O-18:2_20:4) levels with lung cancer(C), phosphatidylcholine (18:1_20:2) levels with lung cancer(D), phosphatidylcholine (O-18:0_16:1) levels with breast cancer(ER+)(E), phosphatidylcholine(O-16:0_20:3) levels with endometrial cancer (endometrioid histology)(F). Each black point representing a SNP is plotted in relation to the effect size of the SNP on the exposure (x-axis) and on the outcome (y-axis) with corresponding standard error bars. The slope of each line corresponds to the causal estimate using IVW (light blue), MR-Egger regression (blue), Simple mode (light green), weighted median (green), and weighted mode (pink) method.
