# Supplementary material for: Unveiling the association between plasma lipid species and pan-cancers: A bidirectional 2-sample Mendelian randomization study
Source: Medicine (Baltimore). 2026 Sep 25;105(39):e50711. doi: 10.1097/MD.0000000000050711 (PMC13619218; doi:10.1097/MD.0000000000050711)
Supplement: Supplementary file 4 [file medi-105-e50711-s009.doc]

**Supplementary Figure S8. Regional association plots for the colocalization analysis of 9 plasma lipid species with the risk of 5 types of cancer (tier 2-3 targets, i.e., PP.H4<0.8).** phosphatidylethanolamine(18:0_20:4) levels with pancreatic cancer(A), phosphatidylcholine(20:4_0:0) levels with lung cancer(B), phosphatidylcholine(O-18:2_20:4) levels with lung cancer(C), phosphatidylcholine (18:1_20:2) levels with lung cancer(D), phosphatidylcholine (O-18:0_16:1) levels with breast cancer(ER+)(E), phosphatidylcholine(O-16:0_20:3) levels with endometrial cancer (endometrioid histology)(F), phosphatidylcholine (18:0_20:4) levels with colorectal cancer(G), sterol ester (27:1/14:0) levels with colorectal cancer(H), sterol ester (27:1/20:3) levels with colorectal cancer(I). The lead SNP is shown as a purple diamond. SNPs within ± 1000 kb of the lead SNP were included; p12=1e-5, prior probability a SNP is associated with both plasma lipid species and cancers.
