# Supplementary material for: Unveiling the association between plasma lipid species and pan-cancers: A bidirectional 2-sample Mendelian randomization study
Source: Medicine (Baltimore). 2026 Sep 25;105(39):e50711. doi: 10.1097/MD.0000000000050711 (PMC13619218; doi:10.1097/MD.0000000000050711)
Supplement: Supplementary file 5 [file medi-105-e50711-s012.doc]

**Supplementary Figure S2. Leave-one-out sensitivity analyses of the SNPs represented the 6 plasma lipid species and 4 types of cancer.** phosphatidylethanolamine(18:0_20:4) levels with pancreatic cancer(A), phosphatidylcholine(20:4_0:0) levels with lung cancer(B), phosphatidylcholine(O-18:2_20:4) levels with lung cancer(C), phosphatidylcholine (18:1_20:2) levels with lung cancer(D), phosphatidylcholine (O-18:0_16:1) levels with breast cancer(ER+)(E), phosphatidylcholine(O-16:0_20:3) levels with endometrial cancer (endometrioid histology)(F). The estimated causal effect is shown for each excluded SNP and the overall estimate using all the SNPs is shown in red. The error bars represent the 95% confidence intervals.
