# Supplementary material for: Unveiling the association between plasma lipid species and pan-cancers: A bidirectional 2-sample Mendelian randomization study
Source: Medicine (Baltimore). 2026 Sep 25;105(39):e50711. doi: 10.1097/MD.0000000000050711 (PMC13619218; doi:10.1097/MD.0000000000050711)
Supplement: Supplementary file 6 [file medi-105-e50711-s013.doc]

**Supplementary Figure S3. Forest plots for 6 plasma lipid species on 4 types of cancer.** phosphatidylethanolamine(18:0_20:4) levels with pancreatic cancer(A), phosphatidylcholine(20:4_0:0) levels with lung cancer(B), phosphatidylcholine(O-18:2_20:4) levels with lung cancer(C), phosphatidylcholine (18:1_20:2) levels with lung cancer(D), phosphatidylcholine (O-18:0_16:1) levels with breast cancer(ER+)(E), phosphatidylcholine(O-16:0_20:3) levels with endometrial cancer (endometrioid histology)(F). Each horizontal solid line reflects the estimated result of a single SNP using the Wald ratio method. The bottom two red lines reflect the overall estimate using all SNPs under the IVW and MR-Egger methods. The red line is completely to the left of 0, indicating a significant negative correlation between exposure factors and outcome. The red line is completely to the right of 0, indicating a significant positive correlation between exposure factors and outcome. If the red line crosses 0, the result is not significant.
