# Supplementary material for: Unveiling the association between plasma lipid species and pan-cancers: A bidirectional 2-sample Mendelian randomization study
Source: Medicine (Baltimore). 2026 Sep 25;105(39):e50711. doi: 10.1097/MD.0000000000050711 (PMC13619218; doi:10.1097/MD.0000000000050711)
Supplement: Supplementary file 7 [file medi-105-e50711-s014.doc]

**Supplementary Figure S5. Leave-one-out sensitivity analyses of the SNPs represented the 14 plasma lipid species and colorectal cancer.** phosphatidylcholine (18:0_20:5) levels with colorectal cancer(A), phosphatidylcholine (18:0_20:4) levels with colorectal cancer(B), phosphatidylcholine (16:1_18:2) levels with colorectal cancer(C), phosphatidylcholine (16:0_20:4) levels with colorectal cancer(D), phosphatidylcholine (14:0_18:2) levels with colorectal cancer(E), phosphatidylcholine (16:0_22:6) levels with colorectal cancer(F), phosphatidylcholine (18:1_18:2) levels with colorectal cancer(G), phosphatidylcholine (16:0_22:5) levels with colorectal cancer(H), phosphatidylcholine (17:0_20:4) levels with colorectal cancer(I), sterol ester (27:1/16:0) levels with colorectal cancer(J), sterol ester (27:1/20:4) levels with colorectal cancer(K), sterol ester (27:1/14:0) levels with colorectal cancer(L), sterol ester (27:1/20:3) levels with colorectal cancer(M), sterol ester (27:1/22:6) levels with colorectal cancer(N). The estimated causal effect is shown for each excluded SNP and the overall estimate using all the SNPs is shown in red. The error bars represent the 95% confidence intervals.
