# Supplementary material for: Unveiling the association between plasma lipid species and pan-cancers: A bidirectional 2-sample Mendelian randomization study
Source: Medicine (Baltimore). 2026 Sep 25;105(39):e50711. doi: 10.1097/MD.0000000000050711 (PMC13619218; doi:10.1097/MD.0000000000050711)
Supplement: Supplementary file 8 [file medi-105-e50711-s015.doc]

**Supplementary Figure S6. Forest plots for 14 plasma lipid species on colorectal cancer.** phosphatidylcholine (18:0_20:5) levels with colorectal cancer(A), phosphatidylcholine (18:0_20:4) levels with colorectal cancer(B), phosphatidylcholine (16:1_18:2) levels with colorectal cancer(C), phosphatidylcholine (16:0_20:4) levels with colorectal cancer(D), phosphatidylcholine (14:0_18:2) levels with colorectal cancer(E), phosphatidylcholine (16:0_22:6) levels with colorectal cancer(F), phosphatidylcholine (18:1_18:2) levels with colorectal cancer(G), phosphatidylcholine (16:0_22:5) levels with colorectal cancer(H), phosphatidylcholine (17:0_20:4) levels with colorectal cancer(I), sterol ester (27:1/16:0) levels with colorectal cancer(J), sterol ester (27:1/20:4) levels with colorectal cancer(K), sterol ester (27:1/14:0) levels with colorectal cancer(L), sterol ester (27:1/20:3) levels with colorectal cancer(M), sterol ester (27:1/22:6) levels with colorectal cancer(N). Each horizontal solid line reflects the estimated result of a single SNP using the Wald ratio method. The bottom two red lines reflect the overall estimate using all SNPs under the IVW and MR-Egger methods. The red line is completely to the left of 0, indicating a significant negative correlation between exposure factors and outcome. The red line is completely to the right of 0, indicating a significant positive correlation between exposure factors and outcome. If the red line crosses 0, the result is not significant.
